# A Saccharide Chemosensor Array Developed Based on an Indicator Displacement Assay Using a Combination of Commercially Available Reagents

**DOI:** 10.3389/fchem.2019.00049

**Published:** 2019-02-25

**Authors:** Yui Sasaki, Zhoujie Zhang, Tsuyoshi Minami

**Affiliations:** Institute of Industrial Science, University of Tokyo, Tokyo, Japan

**Keywords:** saccharide, chemosensor array, phenylboronic acid, indicator displacement assay, colorimetric sensing, regression analysis

## Abstract

Herein, a very simple colorimetric chemosensor array is reported for saccharides (_D_-glucose, _D_-fructose, _D_-xylose, _D_-galactose, _D_-mannose, _L_-rhamnose, and *N*-acetyl-_D_-gluosamine). While various types of chemosensors for saccharides have been investigated extensively to-this-date, tremendous additional efforts are still required on a regular basis for the syntheses of new chemosensors. Complicated syntheses would be a bottleneck, given that artificial receptor-based chemosensing systems are not so popular in comparison to biomaterial-based (e.g., enzyme-based) sensing systems. Toward this end, chemosensor array systems using molecular self-assembled materials can avoid the abovementioned synthetic efforts and achieve simultaneous qualitative and quantitative detection of a number of guest saccharides. Using a practical approach, we focus on an indicator displacement assay (IDA) to fabricate a chemosensor array for colorimetric saccharide sensing. On this basis, 3-nitrophenylboronic acid (3-NPBA) spontaneously reacts with catechol dyes such as alizarin red S (ARS), bromopyrogallol red (BPR), pyrogallol red (PR), and pyrocatechol violet (PV), and yields boronate ester derivatives with color changes. The addition of saccharides into the aqueous solution of the boronate esters induces color recovery owing to the higher binding affinity of 3-NPBA for saccharides, thus resulting in the release of dyes. By employing this system, we have succeeded in discriminating saccharides qualitatively and quantitatively with a classification success rate of 100%. Most importantly, our chemosensor array has been fabricated by only mixing low cost commercially available reagents *in situ*, which means that complicated synthetic processes are avoided for saccharide sensing. We believe this simple colorimetric assay that uses only commercially available reagents can create new, user-friendly supramolecular sensing pathways for saccharides.

## Introduction

**Figure 1:**
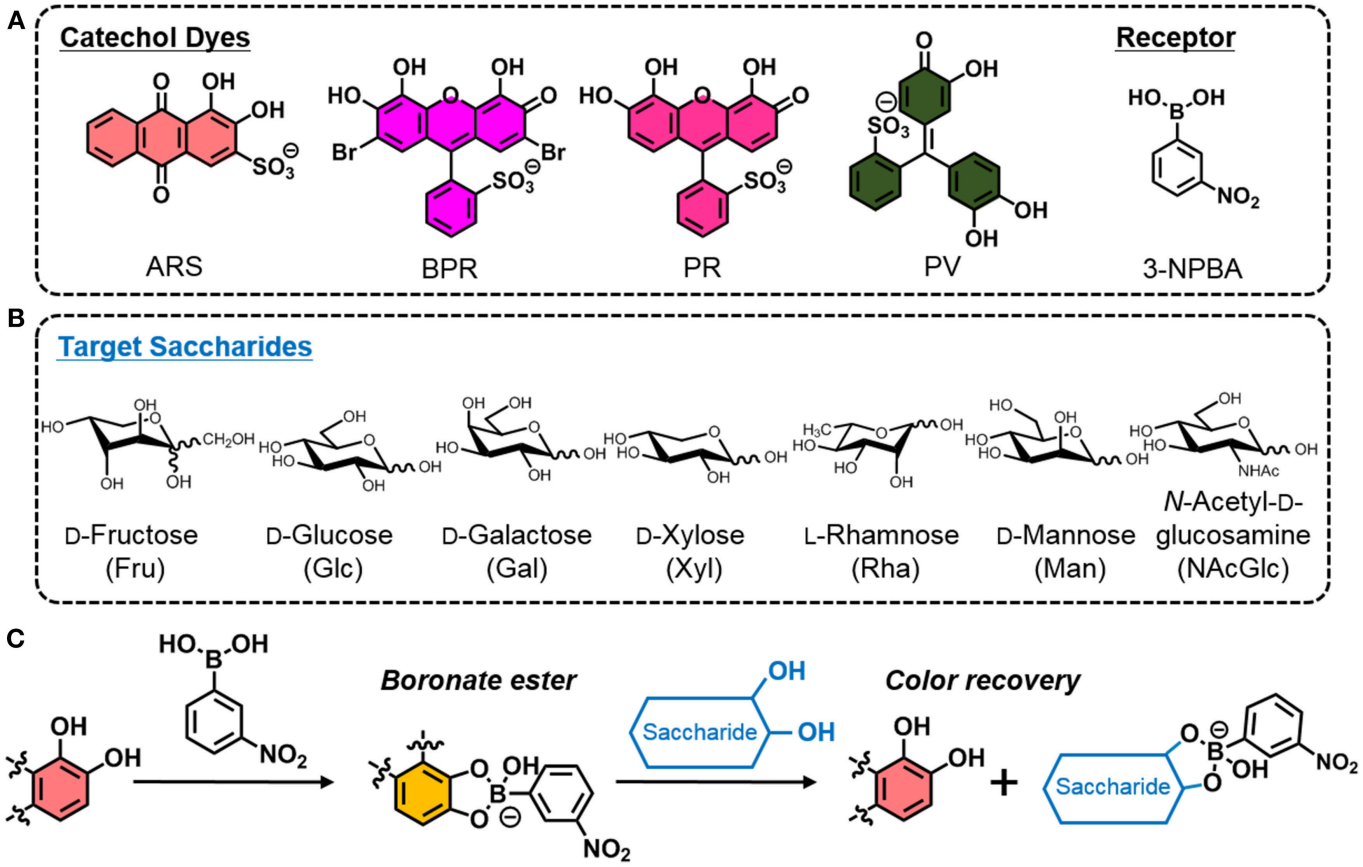
**(A)** Chemical structures of ARS, BPR, PR, PV, and 3-NPBA. **(B)** List of target saccharides. **(C)** Illustrated scheme of the indicator displacement assay utilizing the building blocks (i.e., a catechol dye and 3-NPBA) for the easy preparation of colorimetric sensing.

To-this-date, the analysis of monosaccharides has been proven particularly important in the field of food chemistry because the monitoring of foodstuff quality and the investigation of illegal additions of saccharides into fruit juices or honey are highly required (Tůma et al., 2011). Monosaccharides, such as _D_-(+)-glucose (Glc), _D_-(–)-fructose (Fru), _D_-(+)-xylose (Xyl), _D_-(+)-galactose (Gal), _D_-(+)-mannose (Man), _L_-(+)-rhamnose (Rha) and *N*-acetyl-_D_-(+)-glucosamine (NAcGlc) are generally contained in food or beverages (Martínez Montero et al., 2004), and are conventionally analyzed using instrumental methods (e.g., high-performance liquid chromatography (HPLC) (Schmid et al., 2016) and/or mass spectrometry (MS) (Žídková and Chmelík, 2001) owing to their increased accuracy and reliability. Unfortunately, these methods are associated with increased-costs that incur owing to the use of expensive equipment, relatively complicated procedures, and the necessity of trained personnel. In the efforts to simplify the detection of saccharides, optical chemosensors have been researched extensively (Sun and James, 2015; You et al., 2015). Chemosensors exhibit color and/or fluorescence changes by capturing guest molecules. Accordingly, we can easily recognize the evoked results by simple visual inspection. However, conventional methods used to develop a single chemosensor require a complicated multi step synthesis process (Liu et al., 2015). The latter would prevent the increase of the popularity of the chemosensors in the field of analytical science and industry. In this regard, a molecular self-assembly (Bull et al., 2013) inspired by nature is utilized to prepare saccharide chemsensors *in situ* (Miyaji and Sessler, 2001; Strongin et al., 2001; Sasaki et al., 2017). Herein, we only used a combination of commercially available and inexpensive reagents for the preparation of saccharide chemosensors. This means that 3-nitrophenylboronic acid (3-NPBA) (Hall, 2011) is employed as the saccharide receptor and a catechol dye, such as alizarin red S (ARS), bromopyrogallol red (BPR), pyrogallol red (PR), and pyrocatechol violet (PV), is used as the indicator (Minami et al., 2016) (Figure 1). First, mixing the 3-NPBA and catechol dyes yields boronate esters accompanied by color changes (Springsteen and Wang, 2001; Kubo et al., 2005). Subsequently, a color recovery can be observed by the addition of saccharides because of the dissociation of boronate esters between 3-NPBA and dyes (Ma et al., 2009). This indicator displacement assay (IDA) (Nguyen and Anslyn, 2006), that is used as a sensor array, provides a finger print-like response to saccharides and leads to excellent discrimination results (Maximilian Bojanowski et al., 2017). These results indicate that the smart and appropriate combination of general reagents minimizes synthetic efforts in laboratories, thereby allowing a simplified and easy preparation of supramolecular chemosensors.

## Materials and Methods

### Materials

ARS, Fru, Glc, Xyl, and NAcGlc, were purchased from FUJIFILM Wako Pure Chemical Corporation (Osaka, Japan). Additionally, 3-NPBA, BPR, PR, PV, Gal, Man, and Rha, were purchased from the Tokyo Chemical Industry Co. Ltd. (Tokyo, Japan). Disodium hydrogenphosphate dodecahydrate and sodium dihydrogenephosphate dihydtare were purchased from the Kanto Chemical Co. Inc. (Tokyo, Japan). Diluted solutions used for all photophysical experiments were prepared using Mill-Q water (18.4 MΩ).

### Measurements

UV-vis spectra were measured by a Shimadzu UV-2600 spectrophotometer. UV-vis spectra were recorded within the wavelength range from 350 to 800 nm. Scans were acquired under ambient conditions at 25°C. Saccharide titrations were conducted in a phosphate buffer solution (100 mM) with a pH of 7.4 at 25°C. Titration isotherms were obtained from the changes in the absorption maximum at 455 nm for ARS, 540 nm for BPR, 535 nm for PR, and at 497 nm for PV, respectively. Titration curves, obtained by plotting the change in absorption, were analyzed using non-linear least-squares methods and the equations for the one to one binding model and the IDA model (Hargrove et al., 2010). Equations 1 and 2 were used to fit the UV-vis measurement results,

(1) $$\begin{array}{r} \begin{array}{c} {\left[H\right]}_{t}=\left[H\right]+\;\frac{K_{G}\;\left[H\right]}{1+\;K_{G}\;\left[H\right]}{\left[G\right]}_{t}+\;\frac{K_{I}\;\left[H\right]}{1+\;K_{I}\;\left[H\right]}{\left[I\right]}_{t} \end{array} \end{array}$$

(2) $$\begin{array}{r} \begin{array}{c} A=\;\frac{{\left[I\right]}_{t}}{1+\;K_{I}\;\left[H\right]}\left(\varepsilon_{I}\;b\;+\;\varepsilon_{HI}\;b\;K_{I}\;\left[H\right]\right) \end{array} \end{array}$$

where [G]_t_, [H]_t_, [I]_t_, are the total concentrations of saccharides (as the guests), 3-NPBA (as the host), and for the catechol dyes (as the indicators), respectively. Moreover, *K*_I_ and *K*_G_ are the binding constants of the indicator to the host and the guest to the host, respectively. Furthermore, [H] donates the unknown concentration of the host. The [H] value could be determined using *K*_I_ and *K*_G_, and with the use of the experimentally obtained values [G]_t_, [H]_t_, and [I]_t_. Additionally, ε_I_ and ε_HI_ are the molar absorptivities of the indicator and the complex of the host and the indicator, respectively. Equivalently, *A* and *b* are the saccharide concentration-dependent absorbance and the thickness of the cuvette, respectively.

The array experiment for qualitative and quantitative analyses was performed in 384-well microplates. The fluids [phosphate buffer (100 mM) at pH 7.4, ARS, BPR, PR and PV (40 μM), 3-NPBA (6 mM), and the analyte solutions (100 mM)], were eliminated with a contact-free dispenser as follows. Each experiment was carried out in 24 repetitions. Each well received 90 μL of the buffer solution which contained the catechol dyes and 3-NPBA. Subsequently, 10 μL of analyte solutions or water were dispensed. After this period, the plate was shaken for 3 min. UV-vis spectra were measured by a Biotek SYNERGY H1 microplate reader. The UV-vis spectra were recorded from 400 to 620 nm. The resulting absorption data were applied to the Student's *t*-test to exclude four outlier data points (from the total of 24 repetitions) (Minami et al., 2012). The coefficient of variability of the data was lower than 6%. In the case of qualitative analyses, the obtained data was analyzed using linear discriminant analyses (LDA) (Anzenbacher et al., 2010) without any further pretreatment. The semi quantitative analyses were conducted using LDA after an analysis-of-variance (ANOVA) test. A support vector machine with a principal component analysis preprocessing (PCs = 3) was used for the quantitative assay of the Glc and Fru mixtures.

## Results and Discussion

**Figure 2:**
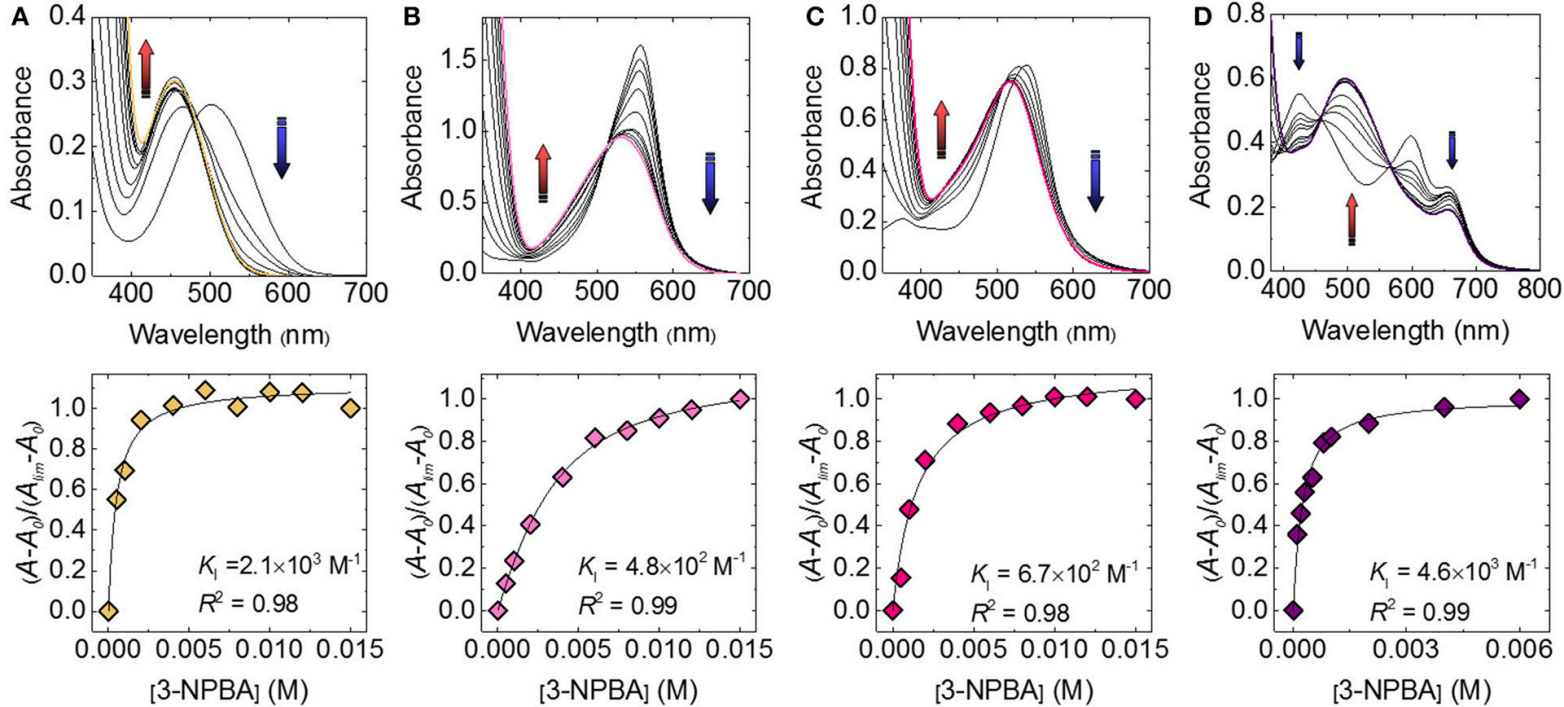
UV-vis spectra of the catechol dye (40 μM) upon the addition of 3-NPBA in a phosphate buffer solution (100 mM) at a pH of 7.4 at 25°C for **(A)** ARS, **(B)** BPR, **(C)** PR, and **(D)** PV.

First, the complexation of catechol dyes and 3-NPBA in a phosphate buffer (100 mM) at pH 7.4 at 25°C was investigated using UV-vis titration experiments. As shown in Figure 2, the absorption spectra of the catechol dyes were shifted as a function of increasing the concentration of 3-NPBA. For example, a significant blue shift (Δλ = 46 nm) was observed in the case of ARS. These responses indicate the formation of the dynamic covalent bond (i.e., boronate esterification), which is identified by fast-atom-bombardment (FAB) mass spectrometry (see the Supplementary Material). The associated constants (*K*_I_s) of these complexes were estimated to be 2.1 × 10^3^ M^−1^ for ARS, 4.8 × 10^2^ M^−1^ for BPR, 6.7 × 10^2^ M^−1^ for PR, and 4.6 × 10^3^ M^−1^ for PV.

**Figure 3:**
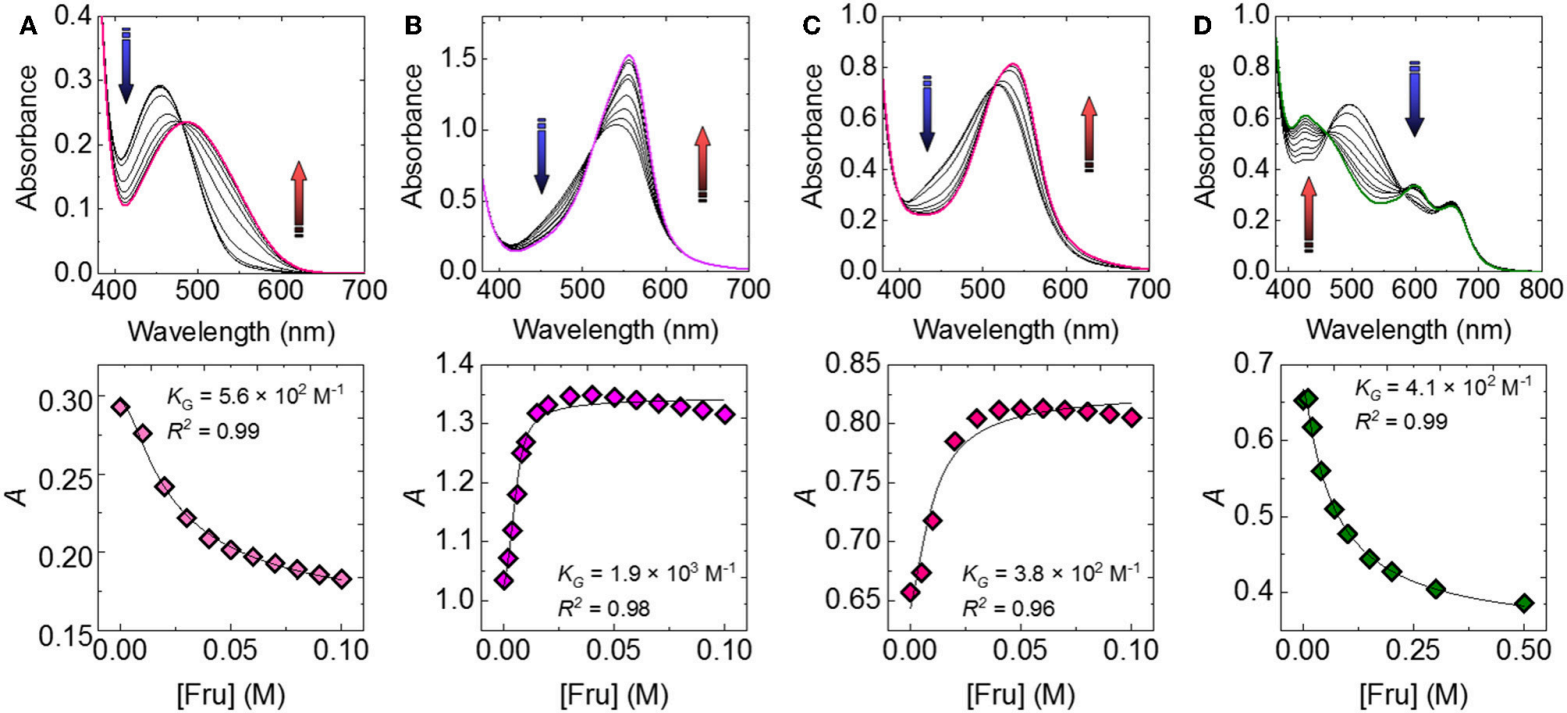
UV-vis spectra of the catechol dye (40 μM)-3-NPBA (6 mM) complex upon the addition of Fru in a phosphate buffer solution (100 mM) at a pH of 7.4 and at 25°C for **(A)** ARS-3-NPBA, **(B)** BPR-3-NPBA, **(C)** PR-3-NPBA, and **(D)** PV-3-NPBA.

**Figure 4:**
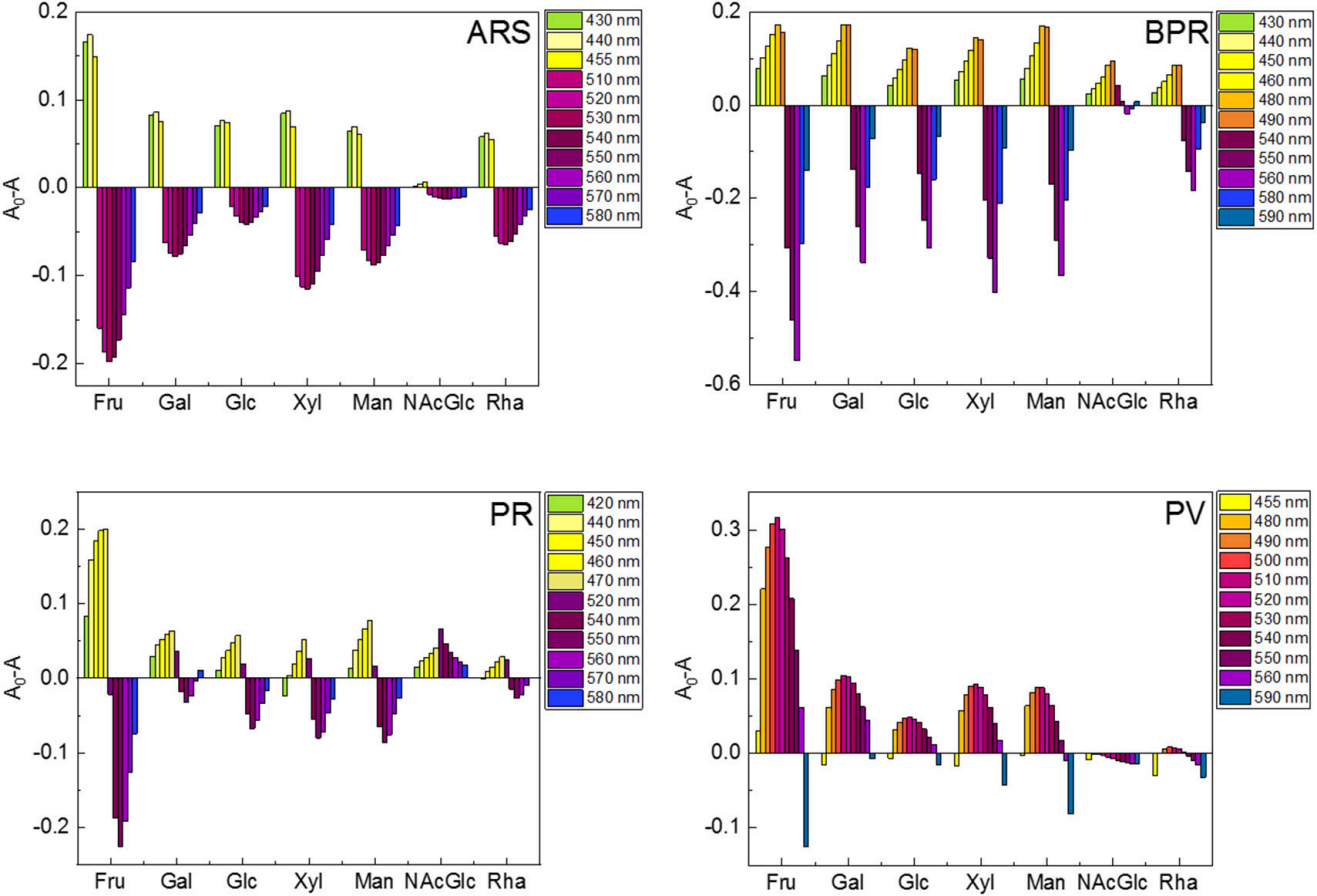
Colorimetric finger print-like response patterns obtained from changes in each absorption wavelength of the catechol dye (40 μM)-3-NPBA (6 mM) complex upon the addition of saccharides (100 mM). **(A)** ARS-3-NPBA, **(B)** BPR-3-NPBA, **(C)** PR-3-NPBA, and **(D)** PV-3-NPBA.

Subsequently, we attempted to detect seven types of monosaccharides (Fru, Glc, Xyl, Gal, Man, Rha, and NAcGlc) which are generally contained in food or beverages. Figure 3 shows the UV-vis titration results of Fru as example. The spectral shift by the incremental Fru concentration was observed to be accompanied by the recovery of the color. The observed recovery suggests that the complexation of 3-NPBA and saccharide occurred on the basis of IDA. Importantly, colorimetric finger print-like responses were obtained by changing the combination of catechol dyes and saccharides (Figure 4). The binding constants between 3-NPBA and saccharides in the presence of catechol dyes are summarized in Table 1. The calculate *K*_G_s were comparable to previously reported colorimetric saccharide chemosensors based on PBAs (Koumoto and Shinkai, 2000; Springsteen and Wang, 2002). From the standpoint of the pattern recognition algorithm, the cross-reactive selectivity is very useful in discriminating various analytes with a high classification accuracy.

**Table 1:** Binding constants (*K*_G_ M^−1^)^a^ on the basis of the indicator displacement assay.

**Dye**	**Fru**	**Gal**	**Glc**	**Xyl**	**Man**	**NAcGlc**	**Rha**
ARS	560 ± 71	110 ± 11	52 ± 4	41 ± 6	<10	<10	17 ± 2
BPR	1900 ± 39	230 ± 24	150 ± 6	110 ± 5	110 ± 13	<10	46 ± 4
PR	380 ± 38	<10	<10	<10	<10	<10	<10
PV	410 ± 49	33 ± 6	16 ± 1	12 ± 2	<10	<10	<10

a *Binding constants were calculated using the change in the UV-vis absorption upon the addition of the analyte in a phosphate buffer solution (100 mM) at a pH of 7.4 at 25°C. All the errors of the binding constants are <19%. Five repetitions were measured for each analyte*.

**Figure 5:**
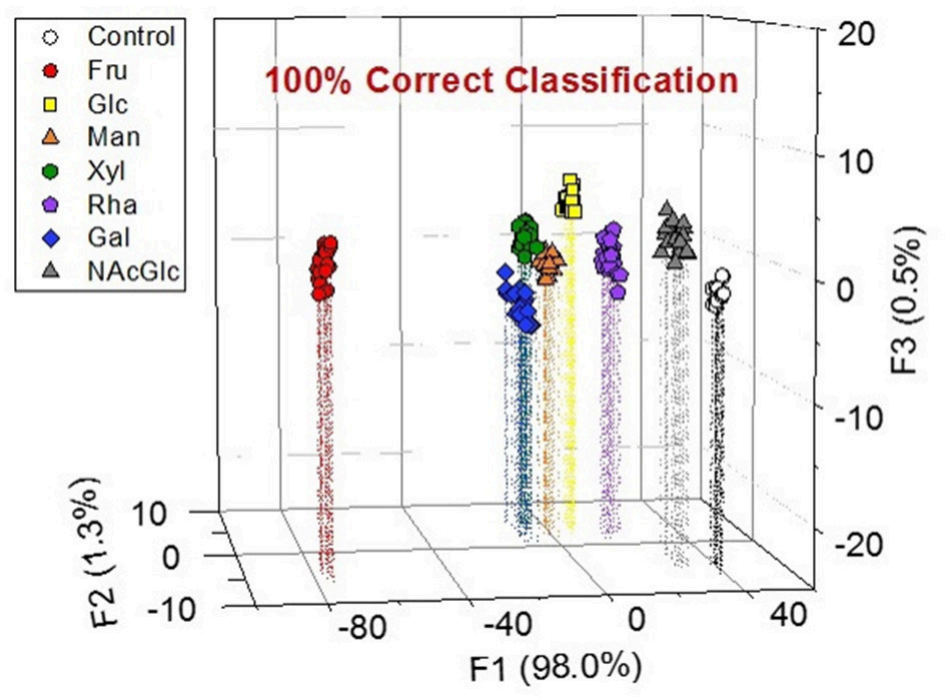
LDA plot for the response of the colorimetric chemosensor array showing the seven types of saccharides (and control) in a phosphate buffer solution at a pH of 7.4 at 25°C. [Saccharide] = 100 mM. Twenty repetitions were measured for each analyte. The cross-validation routine shows a classification success rate of 100%.

Because the finger print-like response encouraged us to fabricate the chemosensor array, we decided to attempt a high-throughput saccharide sensing test. Among the pattern recognition algorithms, we employed LDA as one of the available supervised methods to a) reduce the dimensionality and b) classify the multivariate data. To discriminate analyte patterns, a mathematical model is firstly constructed using a training dataset, which is subsequently evaluated by cross-validation protocols. In our case, a leave-one-out cross-validation protocol (i.e., the jackknife method) was conducted to evaluate the level of correct classification of the observations within the clusters (Anzenbacher et al., 2010). In this assay, 20 repetitions were conducted to confirm reproducibility. We succeeded in discriminating eight clusters (control and seven saccharides, with a total of 160 data points) with a classification success rate of 100% (Figure 5). Interestingly, the position of the Fru cluster is far from the control cluster, most probably owing to the fact that Fru induced the strongest colorimetric response among the tested saccharides. Thus, we can conclude that the LDA plots reflect appropriately the colorimetric responses of the tested saccharides. According to the result of ANOVA (Supplementary Figure 15), the contribution of BPR for discrimination is much higher than the other three dyes. It seems that the relatively high contribution of BPR caused the high F1 value. However, the contribution of the other three dyes is not ignorable. In the absence of ARS, PR or PV, we could not achieve 100% correct classification. Therefore, LDA using four dyes with 3-NPBA is required to discriminate target saccharides.

**Figure 6:**
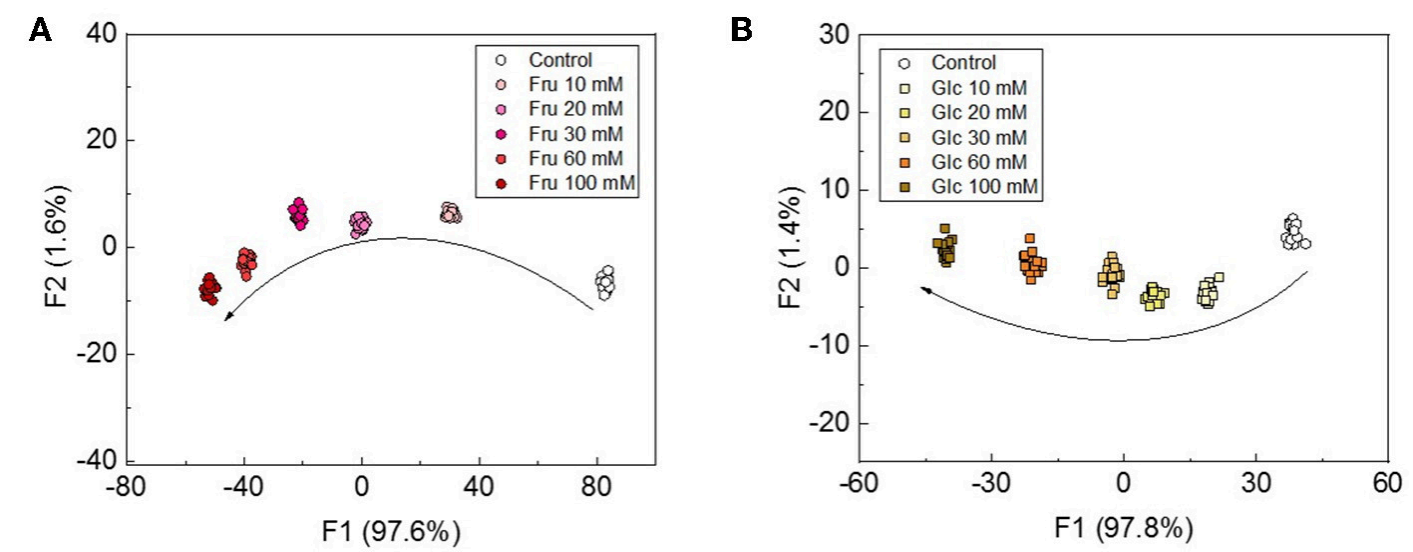
LDA plots for the semi-quantitative assay for **(A)** Fru and **(B)** Glc at the concentration range of 0-100 mM. Twenty repetitions were measured for each concentration.

Although Musto et al., previously reported a qualitative discrimination of saccharides with the use of a colorimetric assay (Musto et al., 2009), quantitative assays for saccharides have not been fully investigated. We thus attempted to apply a semi quantitative assay for Fru and Glc. Beverages, such as fruit juices and wines, generally contain saccharides at concentrations in the range of several tens to hundreds of mM (Han et al., 2016). The LDA was also conducted as the pattern recognition in the semi quantitative assay. This means that the LDA score plots for concentrations in the range of several tens of mM of Fru and Glc were clearly discriminated with classification success rates of 100% (Figure 6). The notable point of the assay is that these cluster positions possess significant trends depending on the saccharide concentrations, which is in agreement with the results of the UV-vis spectroscopic titrations.

From the viewpoint of practical sensing applications, a regression assay for complexed media is necessary. Finally, we demonstrated a quantitative assay for a mixture of Fru and Glc. In this assay, various mixture samples containing eight different concentrations of each saccharide were prepared and were injected in the colorimetric sensor chip. The concentration of Fru was adjusted to gradually decrease, while the concentration of Glc was gradually increased relative to Fru. Owing to the complicated optical responses of the chemosensor array, we employed a support vector machine algorithm (SVM) (Hamel, 2009). The SVM is a powerful analytical method for a quantitative assay, such as the simultaneous prediction of species and concentrations. This method enables the creation of a linear regression line even though an original inset dataset does not show a linear correlation (e.g., analysis of mixed components). The measured UV-vis spectra of chemosensors were analyzed by the SVM, and then unknown concentrations of saccharides in the mixtures were predicted (Supplementary Figure 20). The predicted concentrations (circle dots in Supplementary Figure 20) closely exist on the calibration regression linear line. This indicates that we predicted successfully the saccharide concentrations in the mixtures. The relatively low values of the root-mean-square errors (RMSEs) also indicate the high accuracy of the model and its predictive capacity. To the best of our knowledge, this is the first example that accomplishes colorimetric regression analyses of saccharides in mixtures using only a simple and an appropriate combination of commercially available reagents.

## Conclusion

In summary, we demonstrated the qualitative and quantitative detection of monosaccharides with a simple colorimetric chemosensor array. Owing to the reduced complexity of conventional complicated synthetic methods, the molecular self-assembled system was employed to prepare chemosensors *in situ*. Accordingly, the chemosensor array was fabricated by mixing low-cost, commercially available reagents, such as 3-NPBA, and four types of catechol dyes. The various combinations of these compounds with saccharides generated multi-color response patterns based on the IDA. In the case of the qualitative assay based on the LDA, we succeeded in discriminating of eight distinct groups (control and seven types of saccharides) with a classification success rate of 100%. Furthermore, semi quantitative and quantitative assays for Fru and Glc were conducted and resulted in highly accurate discrimination and prediction. We believe that the simple methods proposed here can be readily conducted by specialists and non-specialists of supramolecular and analytical chemistry, and could contribute to the increase in popularity of chemosensors.

## Author Contributions

YS performed the spectroscopic and the high-throughput array experiments and wrote the manuscript. ZZ performed the spectroscopic experiments and calculated the binding constants. SVM was also performed by ZZ. TM conceived the entire project.

### Conflict of Interest Statement

The authors declare that the research was conducted in the absence of any commercial or financial relationships that could be construed as a potential conflict of interest.
