# Corrigendum: A Saccharide Chemosensor Array Developed Based on an Indicator Displacement Assay Using a Combination of Commercially Available Reagents

**DOI:** 10.3389/fchem.2019.00150

**Published:** 2019-03-22

**Authors:** Yui Sasaki, Zhoujie Zhang, Tsuyoshi Minami

**Affiliations:** Institute of Industrial Science, University of Tokyo, Tokyo, Japan

**Keywords:** saccharide, chemosensor array, phenylboronic acid, indicator displacement assay, colorimetric sensing, regression analysis

In the original article, there was a mistake in Figure 1 as published. The structures given in Figure 1 for pyrocatechol violet and saccharides were incorrect. The corrected Figure 1 appears below.

**Figure 1:**
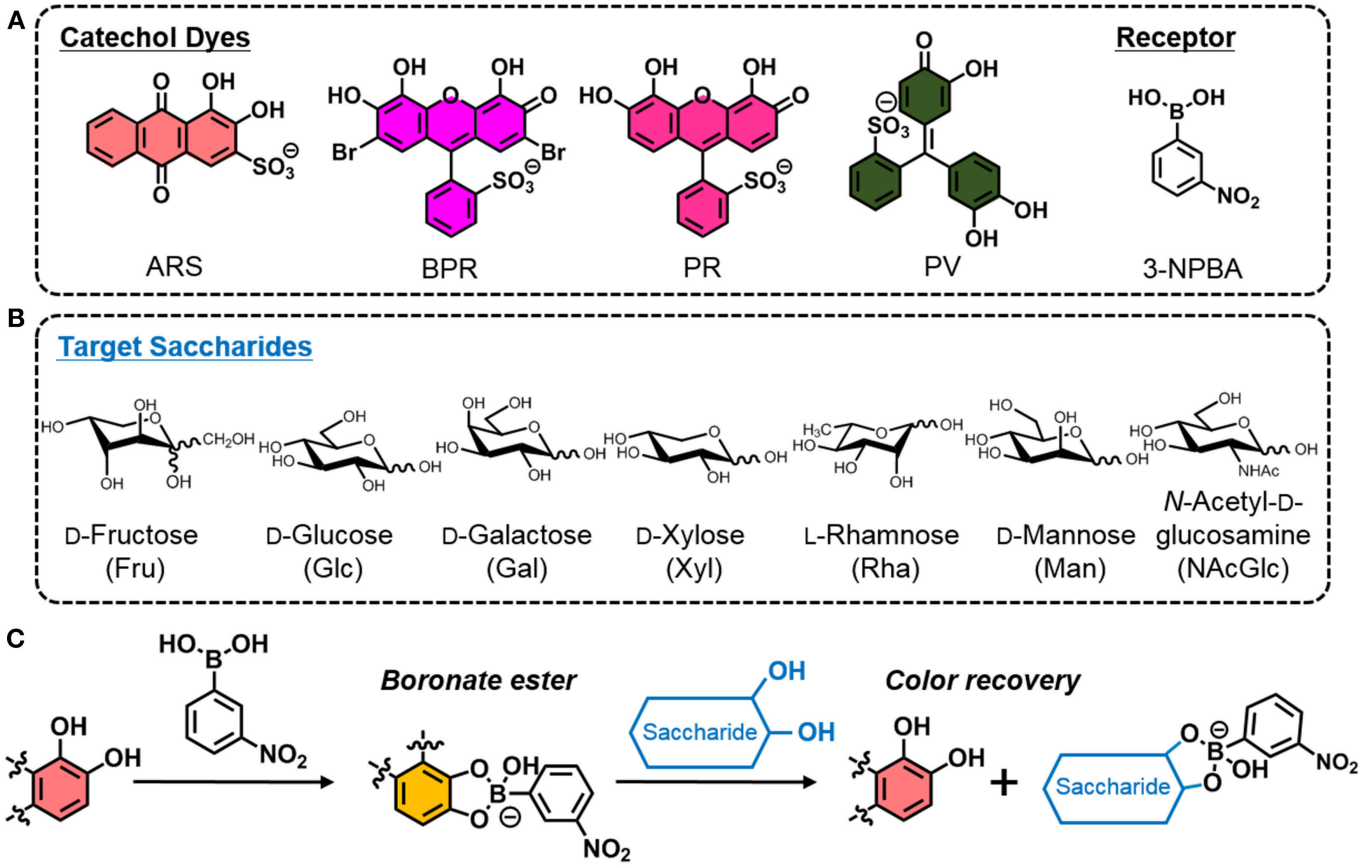
**(A)** Chemical structures of ARS, BPR, PR, PV, and 3-NPBA. **(B)** List of target saccharides. **(C)** Illustrated scheme of the indicator displacement assay utilizing the building blocks (i.e., a catechol dye and 3-NPBA) for the easy preparation of colorimetric sensing.

The authors apologize for this error and state that this does not change the scientific conclusions of the article in any way. The original article has been updated.

