# Supplementary material for: A Saccharide Chemosensor Array Developed Based on an Indicator Displacement Assay Using a Combination of Commercially Available Reagents
Source: Front Chem. 2019 Feb 25;7:49. doi: 10.3389/fchem.2019.00049 (PMC6397832; doi:10.3389/fchem.2019.00049)
Supplement: Supplementary file 1 [file Data_Sheet_1.pdf]

Supplementary Material

A Saccharide Chemosensor Array Developed Based on an Indicator Displacement Assay Using a Combination of Commercially Available Reagents

Yui Sasaki, Zhoujie Zhang and Tsuyoshi Minami*

Institute of Industrial Science, The University of Tokyo, 4-6-1 Komaba, Meguro-ku, Tokyo, Japan

*** Correspondence:**
Tsuyoshi Minami
tminami@iis.u-tokyo.ac.jp

Contents

1. **UV-vis measurements for saccharides** **S2**
2. **FAB MS Analysis** **S6**
3. **Analysis of Variance** **S8**
4. **Linear Discriminant Analysis (LDA)** **S9**
5. **Results of Quantitative Analysis** **S13**

1 UV-vis measurements for saccharides

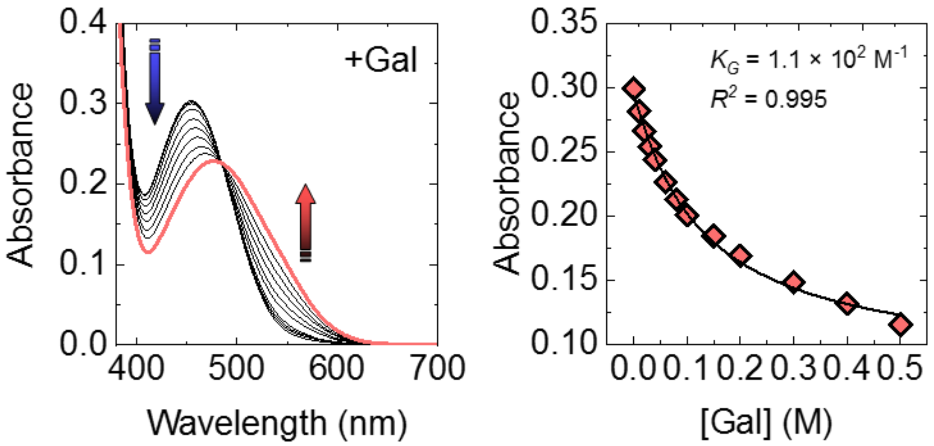

Supplementary Figure 1. UV−vis spectra of the ARS (40 μM)−3-NPBA (6 mM) complex upon the addition of galactose in a phosphate buffer solution (100 mM) at a pH of 7.4 at 25 °C.

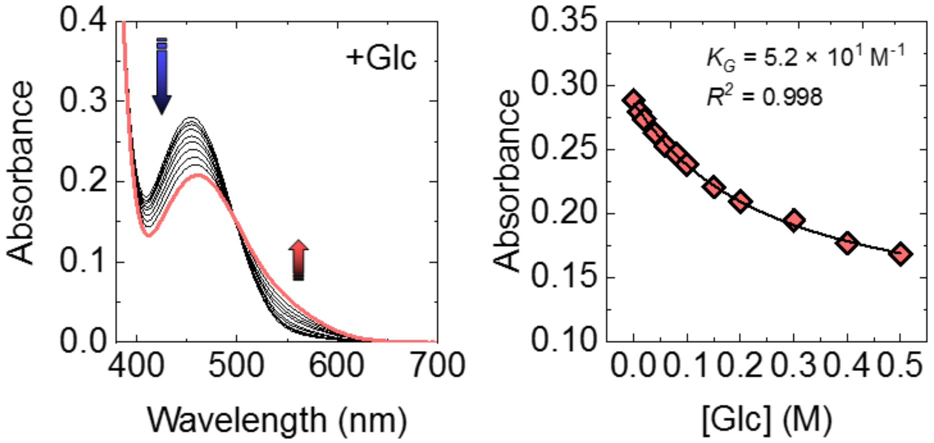

Supplementary Figure 2. UV−vis spectra of the ARS (40 μM)−3-NPBA (6 mM) complex upon the addition of glucose in a phosphate buffer solution (100 mM) at a pH of 7.4 at 25 °C.

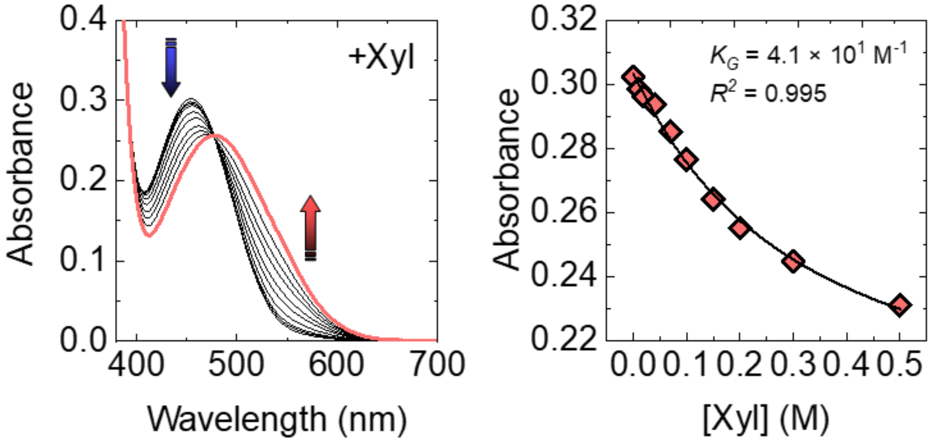

Supplementary Figure 3. UV－vis spectra of the ARS (40 μM)－3-NPBA (6 mM) complex upon the addition of xylose in a phosphate buffer solution (100 mM) at a pH of 7.4 at 25 °C.

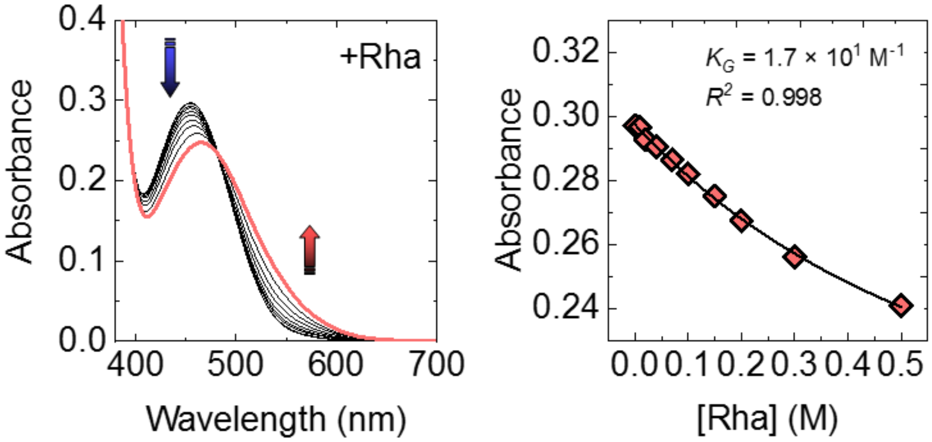

Supplementary Figure 4. UV－vis spectra of the ARS (40 μM)－3-NPBA (6 mM) complex upon the addition of rhamnose in a phosphate buffer solution (100 mM) at at a pH of 7.4 at 25 °C.

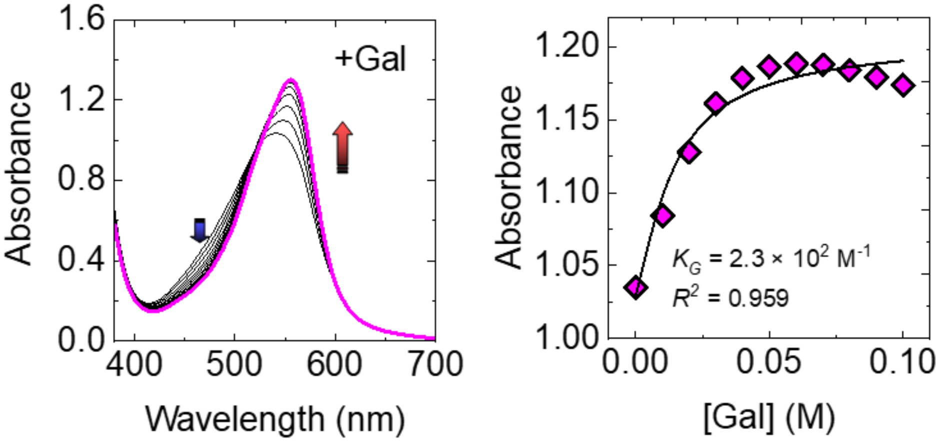

Supplementary Figure 5. UV－vis spectra of the BPR (40 μM)－3-NPBA (6 mM) complex upon the addition of galactose in a phosphate buffer solution (100 mM) at a pH of 7.4 at 25 °C.

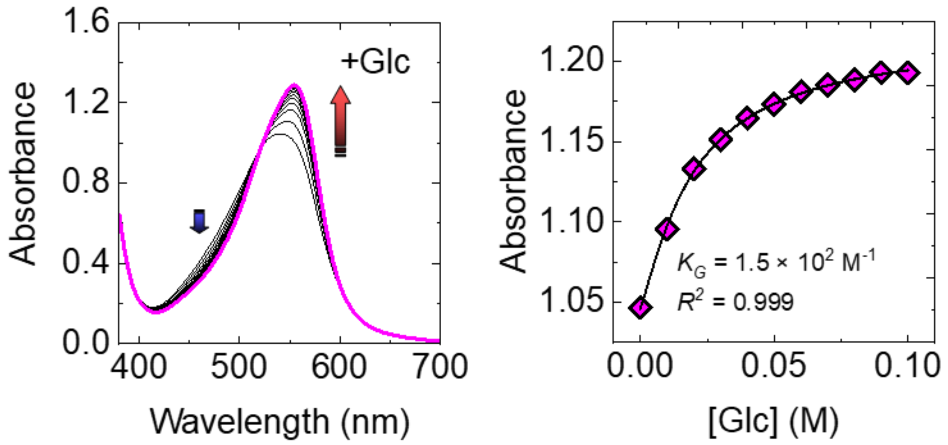

Supplementary Figure 6. UV—vis spectra of the BPR (40 μM)—3-NPBA (6 mM) complex upon the addition of glucose in a phosphate buffer solution (100 mM) at a pH of 7.4 at 25 °C.

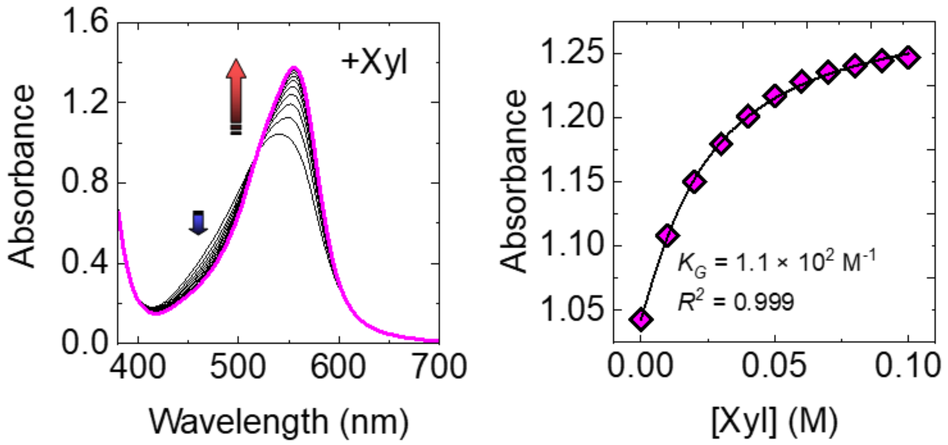

Supplementary Figure 7. UV—vis spectra of the BPR (40 μM)—3-NPBA (6 mM) complex upon the addition of xylose in a phosphate buffer solution (100 mM) at a pH of 7.4 at 25 °C.

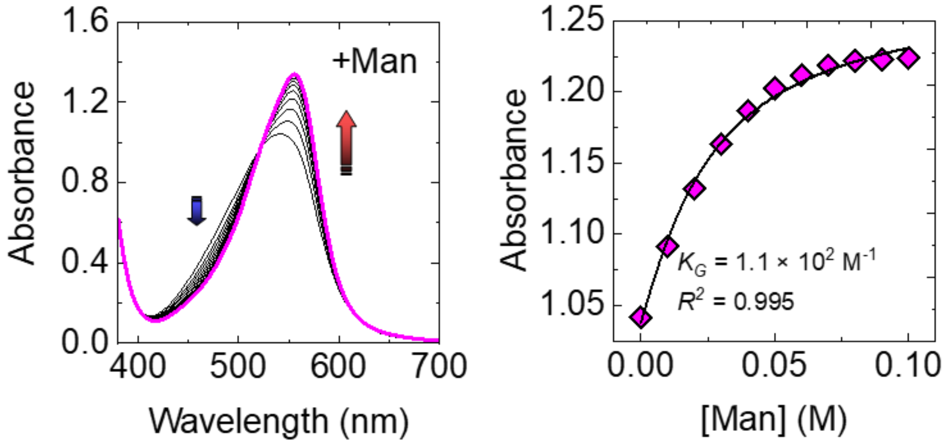

Supplementary Figure 8. UV—vis spectra of the BPR (40 μM)—3-NPBA (6 mM) complex upon the addition of mannose in a phosphate buffer solution (100 mM) at a pH of 7.4 at 25 °C.

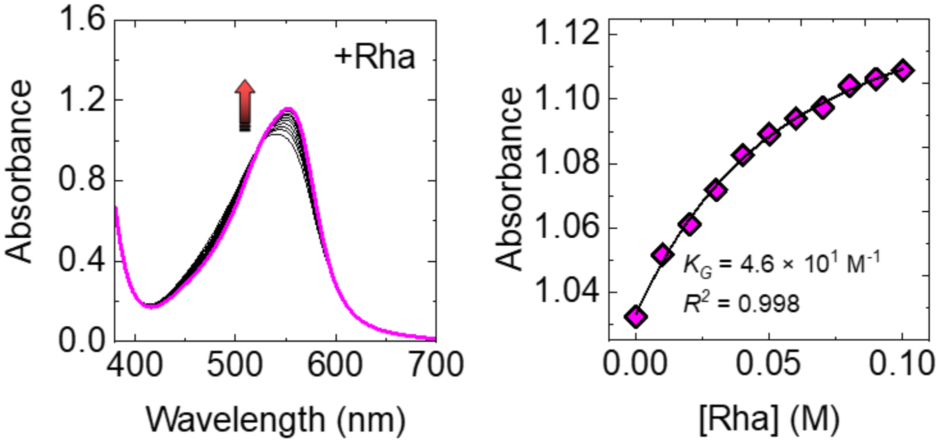

Supplementary Figure 9. UV—vis spectra of the BPR (40 μM)—3-NPBA (6 mM) complex upon the addition of rhamnose in a phosphate buffer solution (100 mM) at a pH of 7.4 at 25 °C.

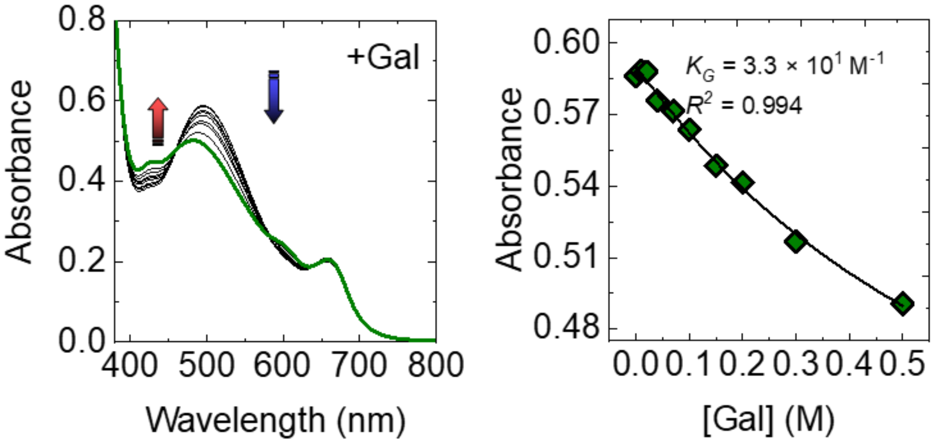

Supplementary Figure 10. UV—vis spectra of the PV (40 μM)—3-NPBA (6 mM) complex upon the addition of galactose in a phosphate buffer solution (100 mM) at a pH of 7.4 at 25 °C.

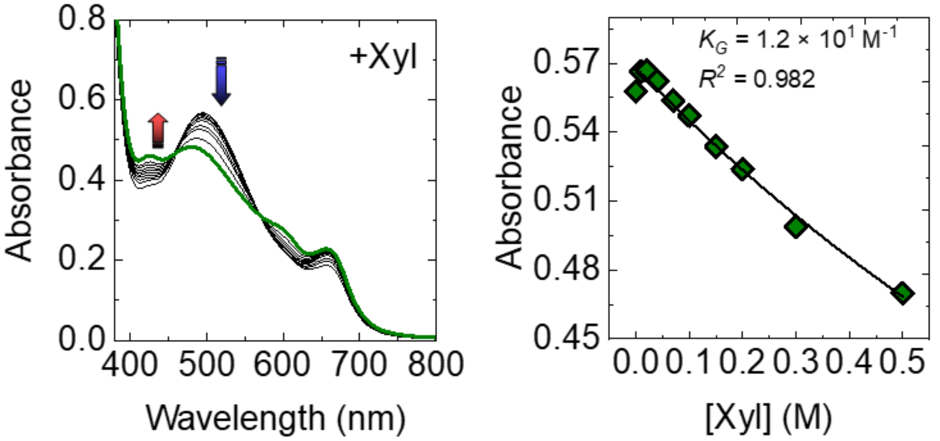

Supplementary Figure 12. UV—vis spectra of the PV (40 μM)—3-NPBA (6 mM) complex upon the addition of xylose in a phosphate buffer solution (100 mM) at a pH of 7.4 at 25 °C.

2 FAB MS Analysis

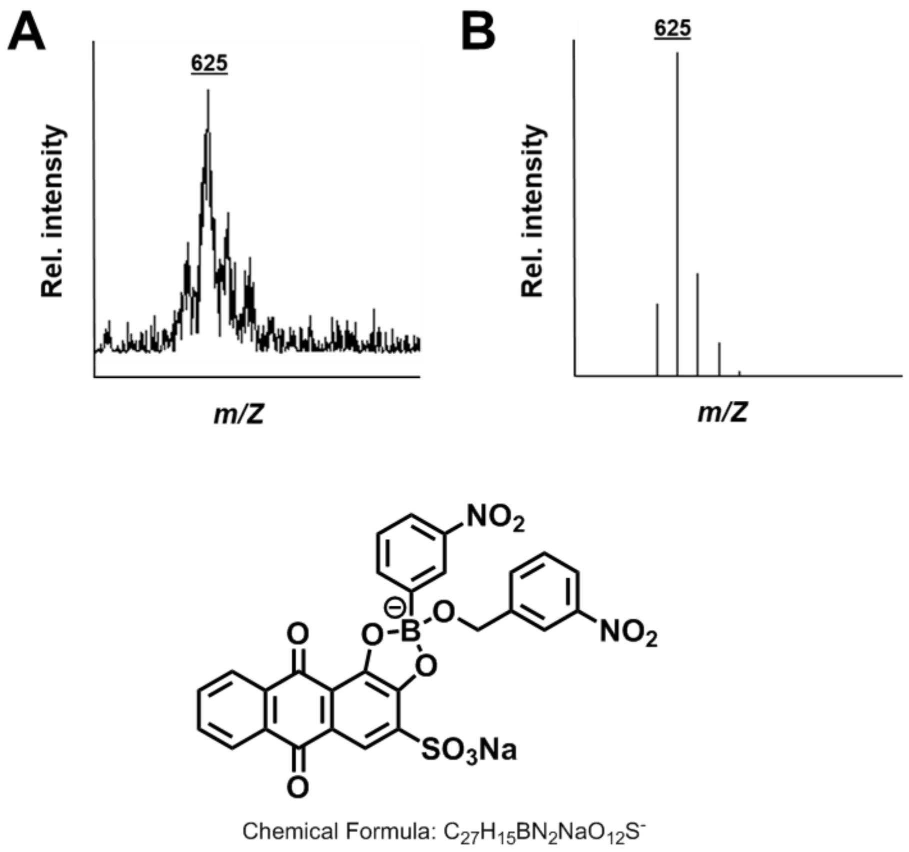

Supplementary Figure 13. (A) FAB MS (negative) spectra of the ARS–3-NBPA complex. (B) Calculated isotope pattern for [ARS-2H_2O+3-NPBA+3-NBA-H]$^-$. Matrix: 3-nitrobenzylalcohol.

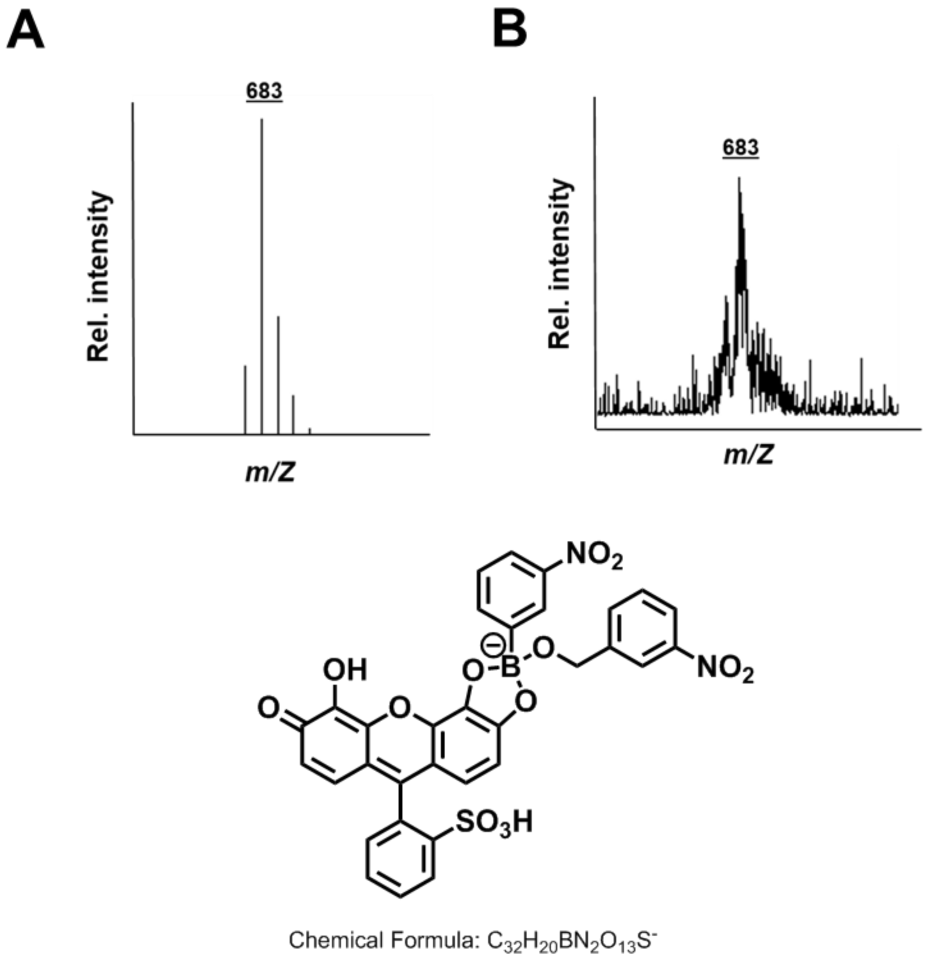

Supplementary Figure 14. (A) FAB MS (negative) spectra of the PR–3-NBPA complex. (B) Calculated isotope pattern for [PR-2H_2O+3-NPBA+3-NBA-H]$^-$. Matrix: 3-nitrobenzylalcohol.

3 Analysis-of-Variance (ANOVA)

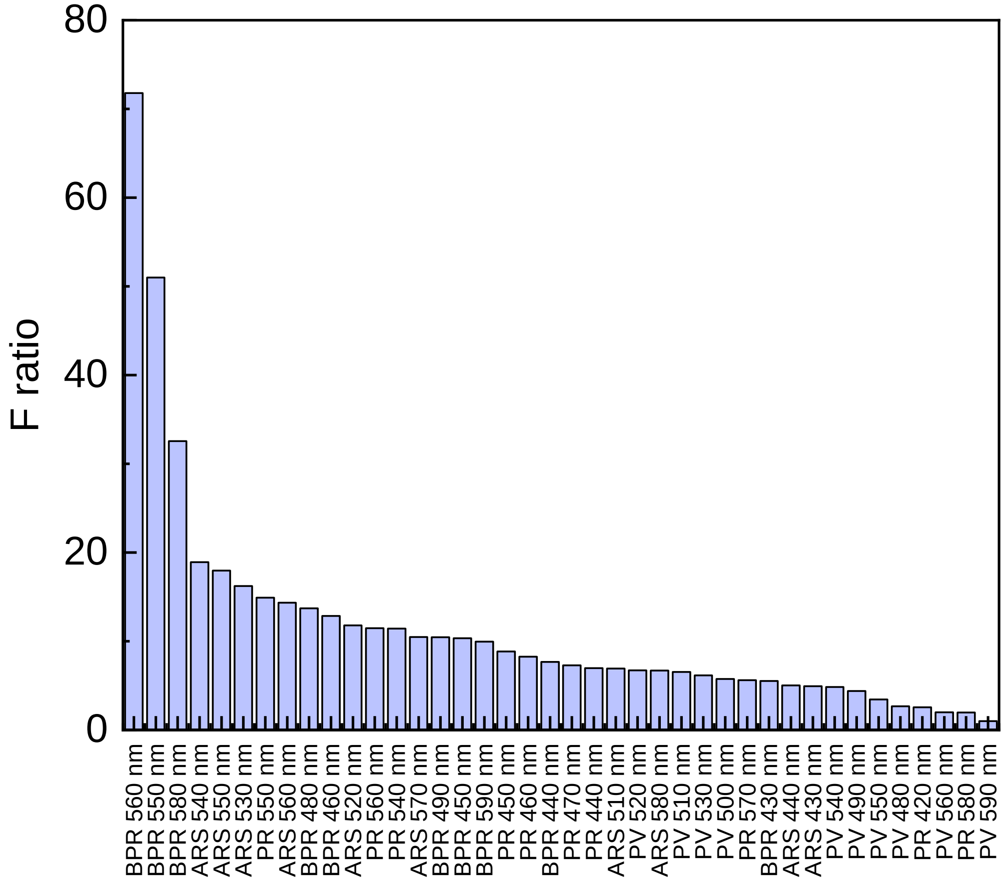

Supplementary Figure 15. One-way ANOVA result of the qualitative analysis.

4 Linear Discriminant Analysis (LDA)

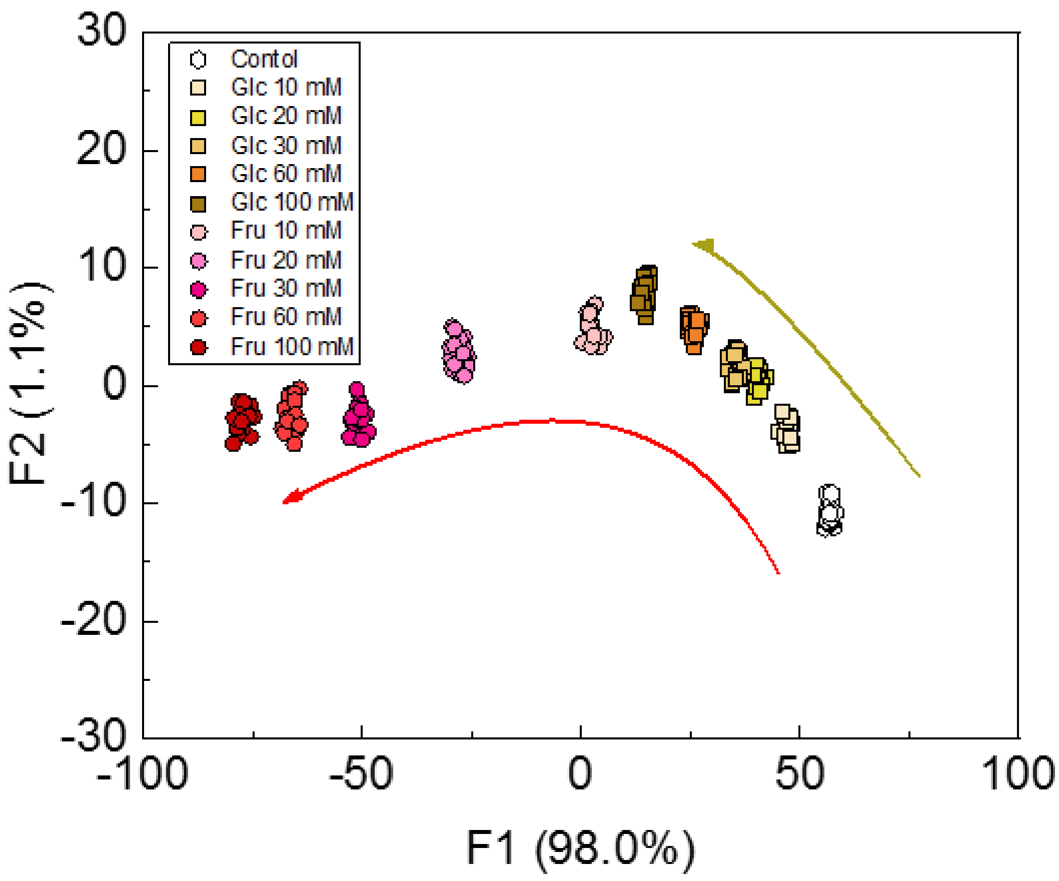

Supplementary Figure 16. LDA plots for the semi-quantitative assay for Fru (○) and Glc (□) at the concentration range of 0－100 mM. Twenty repetitions were measured for each concentration.

Supplementary Table 1 Jackknifed classification matrix of the qualitative assay for Fru and Glc

Jackknifed Classification Matrix	Fru-100mM	Fru-10mM	Fru-20mM	Fru-30mM	Fru-60mM	Glc-100mM	Glc-10mM	Glc-20mM	Glc-30mM	Glc-60mM	control	%correct
Fru-100mM	20	0	0	0	0	0	0	0	0	0	0	100
Fru-10mM	0	20	0	0	0	0	0	0	0	0	0	100
Fru-20mM	0	0	20	0	0	0	0	0	0	0	0	100
Fru-30mM	0	0	0	20	0	0	0	0	0	0	0	100
Fru-60mM	0	0	0	0	20	0	0	0	0	0	0	100
Glc-100mM	0	0	0	0	0	20	0	0	0	0	0	100
Glc-10mM	0	0	0	0	0	0	20	0	0	0	0	100
Glc-20mM	0	0	0	0	0	0	0	20	0	0	0	100
Glc-30mM	0	0	0	0	0	0	0	0	20	0	0	100
Glc-60mM	0	0	0	0	0	0	0	0	0	20	0	100
control	0	0	0	0	0	0	0	0	0	0	20	100
Total	20	20	20	20	20	20	20	20	20	20	20	100

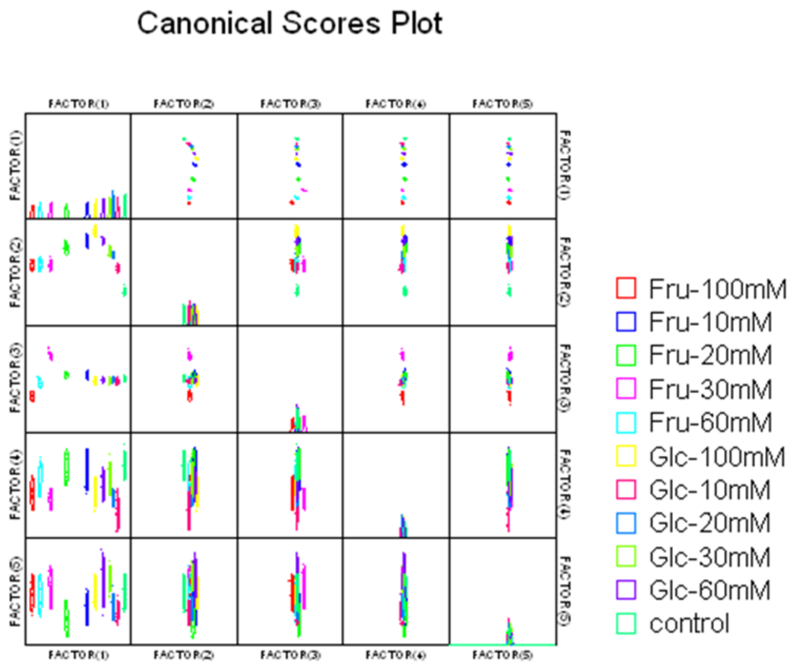

Supplementary Figure 17. Canonical score plot of the qualitative assay for Fru and Glc.

Supplementary Table 2 Jackknifed classification matrix of the qualitative assay for Fru

Jackknifed Classification Matrix							
	Fru-100mM	Fru-10mM	Fru-20mM	Fru-30mM	Fru-60mM	control	%correct
Fru-100mM	20	0	0	0	0	0	100
Fru-10mM	0	20	0	0	0	0	100
Fru-20mM	0	0	20	0	0	0	100
Fru-30mM	0	0	0	20	0	0	100
Fru-60mM	0	0	0	0	20	0	100
control	0	0	0	0	0	20	100
Total	20	20	20	20	20	20	100

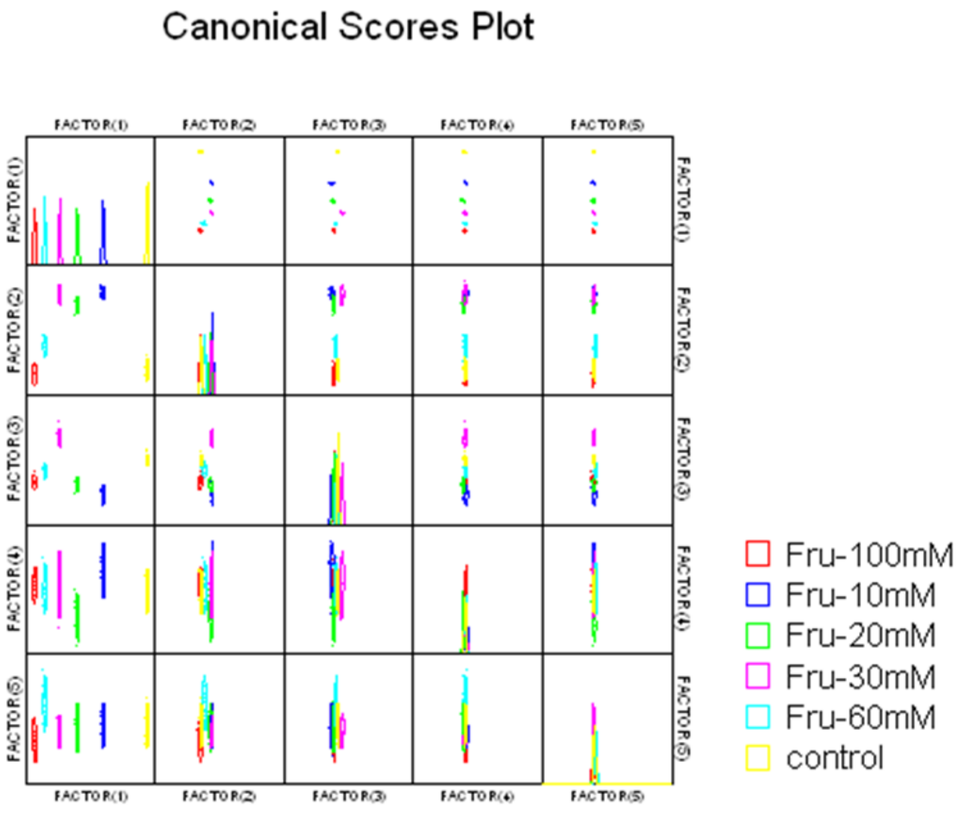

Supplementary Figure 18. Canonical score plot of the qualitative assay for Fru.

Supplementary Table 3 Jackknifed classification matrix of the qualitative assay for Glc

Jackknifed Classification Matrix							
	Glc-100mM	Glc-10mM	Glc-20mM	Glc-30mM	Glc-60mM	control	%correct
Glc-100mM	20	0	0	0	0	0	100
Glc-10mM	0	20	0	0	0	0	100
Glc-20mM	0	0	20	0	0	0	100
Glc-30mM	0	0	0	20	0	0	100
Glc-60mM	0	0	0	0	20	0	100
control	0	0	0	0	0	20	100
Total	20	20	20	20	20	20	100

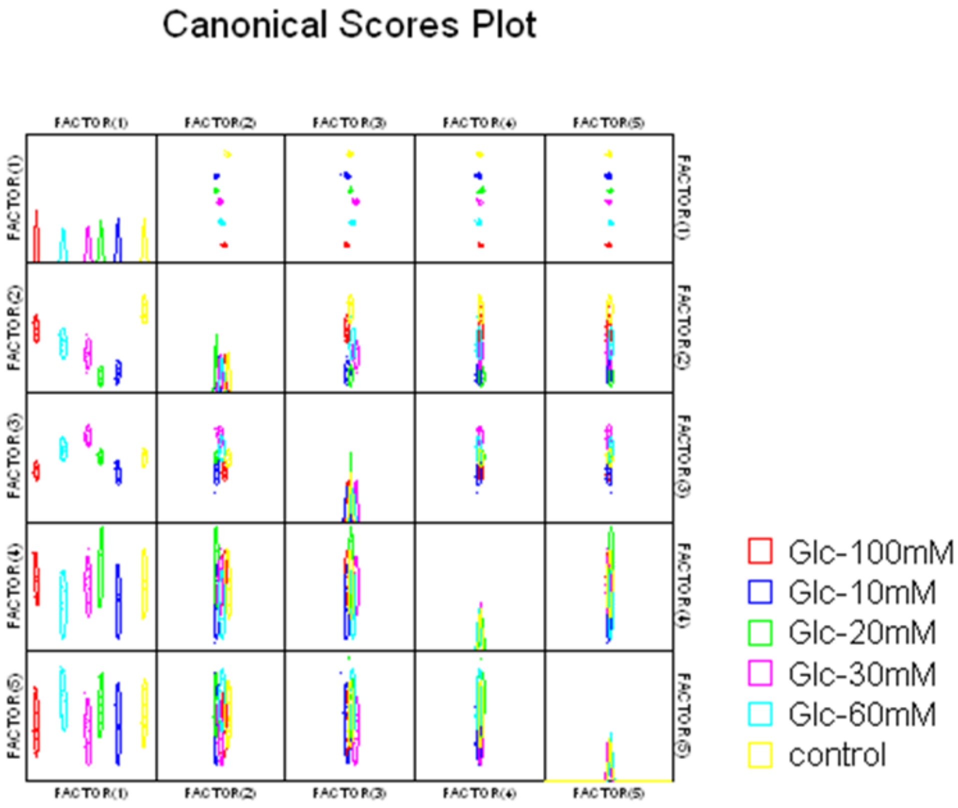

Supplementary Figure 19. Canonical score plot of the qualitative assay for Glc.

5 Results of Quantitative Analysis

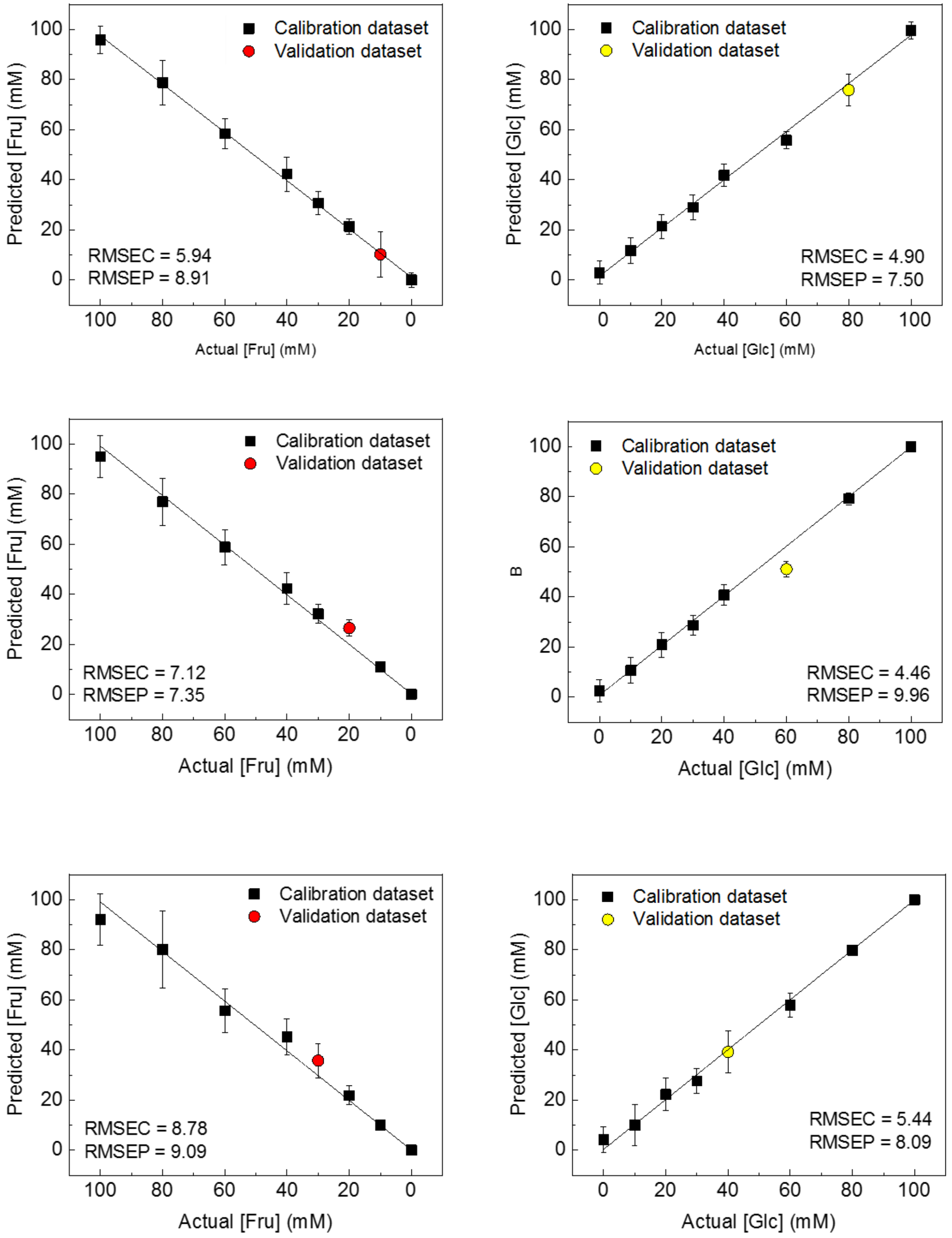

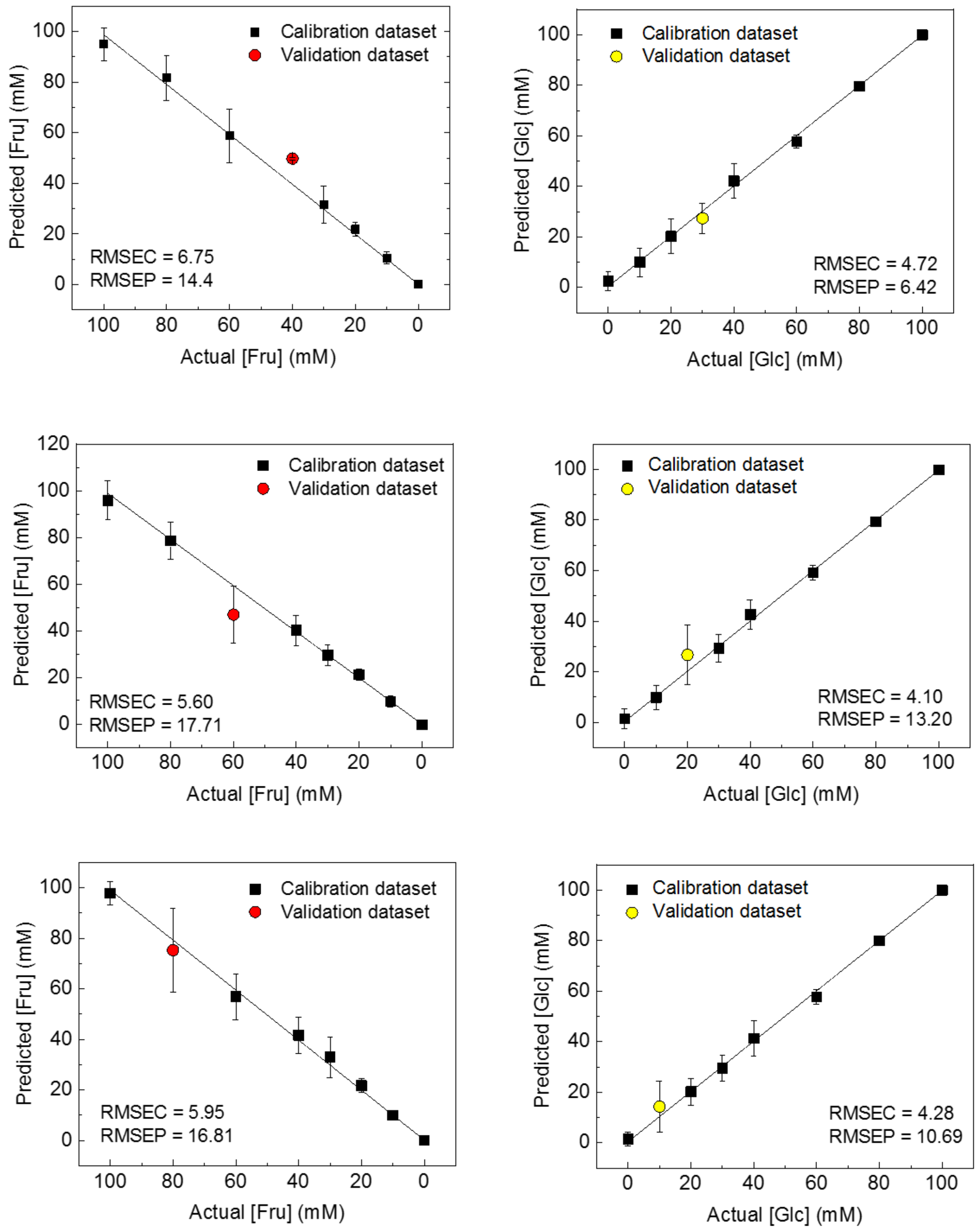

Supplementary Figure 20 SVM regression results used for quantitative analyses of Fru and Glc mixtures. The values of the root-mean-square errors of calibration (RMSEC) and prediction (RMSEP) (shown as insets) attest to the high accuracies of the model and its predictive capacity.
